# Retrospective observational study of patient outcomes with local wound infusion vs epidural analgesia after open hepato-pancreato-biliary surgery

**DOI:** 10.1186/s12871-022-01563-2

**Published:** 2022-01-18

**Authors:** A. C. Jackson, K. Memory, E. Issa, J. Isherwood, P. Graff-Baker, G. Garcea

**Affiliations:** 1 Leicester Medical School, University of Leicester, Leicester, United Kingdom; 2 HPB Department, University Hospitals Leicester NHS trust, Leicester, United Kingdom; 3 Anaesthetics Department, University Hospitals Leicester NHS trust, Leicester, United Kingdom

**Keywords:** Wound infusion catheter, HPB surgery, Epidural

## Abstract

**Background:**

Epidural analgesia is conventionally used as the mainstay of analgesia in open abdominal surgery but has a small life-changing risk of complications (epidural abscesses or haematomas). Local wound-infusion could be a viable alternative and are associated with fewer adverse effects.

**Methods:**

A retrospective observational analysis of individuals undergoing open hepato-pancreato-biliary surgery over 1 year was undertaken. Patients either received epidural analgesia (EP) or continuous wound infusion (WI) + IV patient controlled anaesthesisa (PCA) with an intraoperative spinal opiate. Outcomes analyzed included length of stay, commencement of oral diet and opioid use.

**Results:**

Between Jan 2016- Dec 2016, 110 patients were analyzed (WI *n=35*, EP *n=75*). The median length of stay (days) was 8 in both the WI and EP group (*p=0.846*), the median time to commencing oral diet (days) was 3 in WI group and 2 in EP group (*p=0.455*). There was no significant difference in the amount of oromorph, codeine or tramadol (mg) between WI and EP groups (*p*=*0.829*, *p=0.531, p=0.073,* respectively).

**Conclusions:**

Continuous wound infusion + IV PCA provided adequate analgesia to patients undergoing open hepato-pancreato-biliary surgery. It was non-inferior to epidural analgesia with respect to hospital stay, commencement of oral diet and opioid use.

## Introduction

Open hepato-pancreato-biliary procedures are known to be associated with high-levels of post-operative pain [1]. Inadequate management of post-operative pain can contribute to the neuroendocrine stress response and has historically been associated with increased complications and prolonged length of stay, adversely affecting overall patient outcome [1, 2].

Epidural analgesia (EP) is currently the mainstay of pain control following open hepato-pancreato-biliary procedures in most centres [3]. It is an effective modality which has superseded systemic intravenous opioids due to superior analgesic properties and fewer opioid-related side effects [4]. Although EP has a greater analgesic efficacy, it can cause life-threatening or life-changing (albeit rare) complications, such as epidural abscess or haematoma. Despite these complications being rare, EP-associated post-operative hypotension is a relatively common complication [4] and subsequent fluid-resuscitation has been linked to acute kidney injury and fluid overload [1, 5].

Furthermore, there are relative contraindications to consider when using epidural analgesia, including that of previous spinal surgery, uncorrected hypovolaemia and coagulopathy [6]. The latter requires special consideration for particular procedures, such as liver resections [6, 7], with one study demonstrating that in 15% of patients, epidural catheter removal was delayed by 1-3 days due to coagulopathy [6]. The increased risk of coagulopathy in this subgroup of patients could suggest that epidural analgesia may not be optimally safe for patients undergoing liver resections [7].

In recent years, the use of local anaesthetic infiltration via wound catheters (WI) placed in the abdominal wall has been explored in a variety of procedures [4], including open nephrectomies; in which it was found to be an effective alternative [8]. As well as directly blocking transmission from nociceptors, local wound infusion can further contribute to analgesia via inhibiting local inflammation, which itself leads to increased sensitivity and hyperalgesia, thus contributing to post-operative pain [9]. Numerous studies have since confirmed its analgesic efficacy in liver resections [10, 11], but more evaluation is needed of the direct comparison between WI and EP modalities.

The majority of patients receiving a wound catheter concomitantly receive IV patient-controlled analgesia and, where possible, intrathecal opioid administration pre-operatively. Some studies have found that intrathecal analgesia has superior analgesic properties to epidural analgesia, in both speed of onset and sacral nerve coverage [12], whilst simultaneously having fewer complications than that of epidural analgesia [12, 13], including reduced rates of post-operative hypotension, excessive intravenous fluid administration and overall length of stay [7, 13, 14].

The aim of this retrospective analysis was to directly compare patient outcomes and additional opioid use after open hepato-pancreato-biliary procedures over a 1-year period. As WI itself is safe, easy to administer and has fewer side-effects than EP (as does spinal analgesia) [5], the combination should be considered as an alternative modality for post-op analgesia if opioid exposure and patient outcomes do not differ from that of EP alone.

## Material and Methods

Data were collected retrospectively from notes of patients who underwent open hepato-pancreato-biliary procedures, through a standardised roof-top incision, at the Leicester General Hospital from January 2016 to December 2016. Operations included predominantly liver resections, pancreaticoduodenectomies and distal pancreatectomies +/- splenectomy. Each liver resection was defined as either major or minor, with minor defined as <3 segments resected and major defined as ≥3 segments resected. All epidural analgesia given was a combination of fentanyl and bupivacaine. All wound catheter infiltration was with 0.25% bupivacaine.

A standardized data collection spreadsheet was used, with variables recorded including patient characteristics (age, gender, BMI and ASA grade), anaesthetic and intraoperative details, postoperative analgesia, post-operative complications, time to eating and drinking and length of hospital stay (both HDU [high-dependency unit] and overall). Intraoperative data included blood loss, operation duration and units transfused. Parameters such as intra-operative blood pressure, fluid administration and post-operative nausea and vomiting (PONV) were not analysed in either arm due to lack of data in patient notes.

The date of the surgery was classed as day 0. Hypertension, ischaemic heart disease, chronic kidney disease, chronic obstructive pulmonary disease, cerebrovascular disease, diabetes and immunosuppression were all considered major co-morbidities, and the presence of any of these in each patient was recorded. No patients were known to have chronic pain issues prior to their surgery.

Within the WI arm, comparisons of patients who had a pre-operative spinal injection and those that didn’t were also carried out. Intraoperative bleeding, operation duration, overall complications and post-operative oral and IV analgesia were analysed. Intraoperative details recorded include operation duration (mins), blood loss (ml) and units transfused. All patients were part of an enhanced recovery protocol post-operatively. Patients were non-randomised, and the choice of WI or EP was dependent on surgeon/anaesthetic preferred practice, but specific reasons for choice of analgesic modality were not recorded in patient notes.

As well as length of stay, monitoring within both the HDU and ward setting included commencement of oral diet, classification of post-operative complications (via Clavien-Dindo classification [15]) and recording total amount of opioids given post-operatively (through all routes). Oral opioids noted included Oromorph, Codeine and Tramadol (mg), as well as those administered intravenously, intrathecally or via epidural infusion (morphine sulphate {mg}, diamorphine {mcg} or fentanyl {mcg}, respectively). The total amount of each opioid given (in mg or mcg) was determined in both the epidural arm and the wound catheter arm, as a marker of post-operative pain control.

Epidural failure was defined by the use of IV-PCA within 48 hours in patients in whom an epidural had been placed.

Although data on peri-operative blood pressure, fluid balance and IV fluid administration was not collated in this study, class I and II complications according to Clavien-Dindo classification [15] would include use of intravenous fluids or pharmaceuticals to treat hypotension.

Postoperative days were defined as beginning and ending at 08.00 h each day.

All data were entered into a database using Microsoft Excel ‘19. Data were analysed using GraphPad Prism 8. With regards to statistical analysis, Mann-Whitney U tests, Independent Samples t-Tests, Chi-Squared tests and one-way ANOVA tests were used where appropriate. Data are presented as medians (ranges) except where stated otherwise.

## Results

**Fig. 1:**
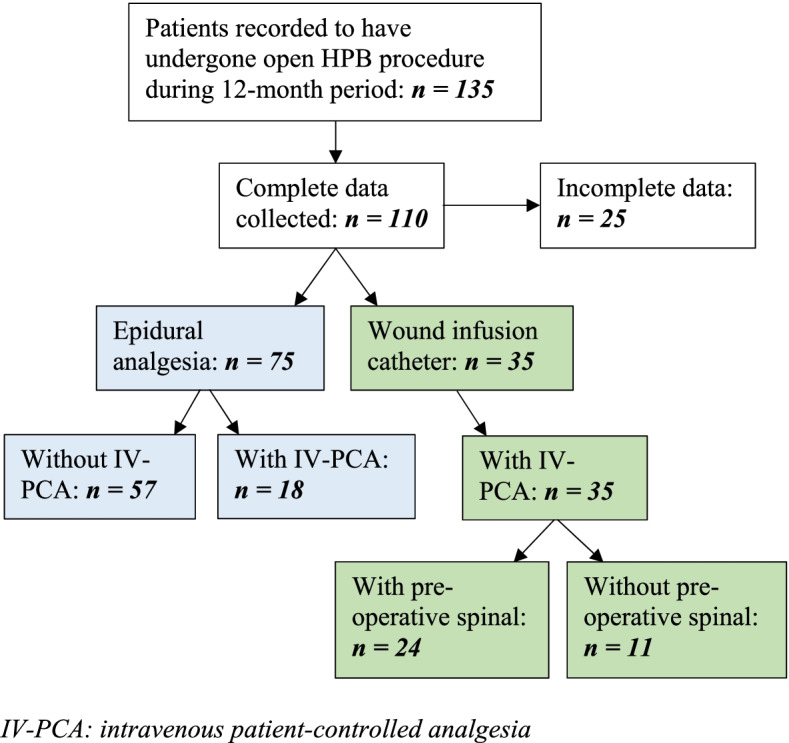
Flow diagram demonstrating patient arms analysed and compared in this study

During the 12-month period, 135 patients were recorded to have undergone an open HPB procedure. 110 of these (81.4%) had complete data to collect. 75 of these patients received EP for their mainstay of analgesia, with 35 patients receiving local wound infusion (WI) with bupivacaine 0.25%. All patients with WI additionally received post-operative patient-controlled analgesia (PCA) (n=35), with 24/35 patients also receiving pre-operative spinal analgesia (diamorphine, mcg). 18 (24%) of EP patients concurrently received post-operative PCA within the first 48 hours, which was defined as epidural failure (Fig 1).

### Patient characteristics

There was no statistically significant difference between patient groups with regards to age, BMI or ASA grade (Table 1). No patients were known to have chronic pain issues prior to their surgery.

**Table 1:** Baseline characteristics and intraoperative details including procedure, operation duration, blood loss and units transfused

Patient characteristics	WI *n =* ***35***	EP *n =* ***75***	***P-value***
Age (years), median (IQR)	65 (58-72)	67 (61-74)	*0.249*
BMI, median (IQR)	27 (24.4-31)	26.1 (24-29)	*0.195*
ASA physical status, *n (%)*			*0.375*
I	4 (11.4)	2 (2.7)	
II	18 (51.4)	60 (80.0)	
III	13 (37.1)	13 (17.3)	
**Type of surgery,** *n (%)*			
**Liver resection**	**17** (48.6)	**43** (57.3)	*0.390*
Section/segment/metastectomy	9 (25.7)	27 (36.0)	*0.284*
≥3 segments	8 (22.9)	16 (21.3)	*0.896*
**Pancreas**	**15** (42.9)	**27** (36.0)	*0.491*
Whipples (classic)	2 (5.7)	11 (14.7)	*0.176*
Whipples (PPPD)	5 (14.2)	8 (10.7)	*0.584*
Distal pancreas/spleen	8 (22.9)	8 (10.6)	*0.091*
**Other**	**3** (8.6)	**5** (6.7)	*0.720*
**Intraoperative data**			
Operation duration (mins) median (IQR)	210 (150-260)	210 (127.5-310)	*0.838*
Blood loss (ml), median (IQR)	300 (150-500)	250 (150-500)	*0.444*
Units transfused			
1	0	4	*0.164*
2	2	4	*0.935*
3	0	0	
4	0	1	*0.493*

*BMI* Body mass index, *ASA* American Society of Anaesthesiologists

### Intraoperative details

With regards to the categorized procedures, there was no statistically significant difference in the number of liver or pancreatic resections between the WI and EP groups (Table 1). Those operations classified as ‘other’ (Table 1) included resection of distal antrum, excision of a choledochal cyst, left adrenalectomy and bile duct reconstruction.

When analyzing intraoperative details, there was no statically significant difference with regards to operation duration, blood loss or units transfused, between both patient groups (Table 1).

Parameters such as intra-operative blood pressure, fluid administration and post-operative nausea and vomiting (PONV) were not analysed in either arm due to lack of data in patient notes.

### Post-operative recovery

Post-operative complications were classified according to the Clavien-Dindo classification, with the majority of patients falling into category I (Table 2). There was no statistically significant difference in any particular class of complications between each group (Table 2).

**Table 2:** Highest grade of complication, as per Clavien-Dindo classification, and rate of readmission for both WI and EP groups

	WI ***n =*** 35	EP ***n = 75***	*P-value*
**Highest grade of complication** *n (%)*			
I	27 (77.1)	51 (66.0)	*0.325*
II	4 (11.4)	21 (28.0)	*0.053*
IIIa	0	0	
IIIb	2 (5.7)	2 (2.7)	*0.426*
IVa	0	0	
IVb	2 (5.7)	1 (1.3)	*0.189*
V	0	0	
**Readmission within 2 weeks** *n (%)*	1 (2.9)	6 (8.0)	*0.303*

**Fig. 2:**
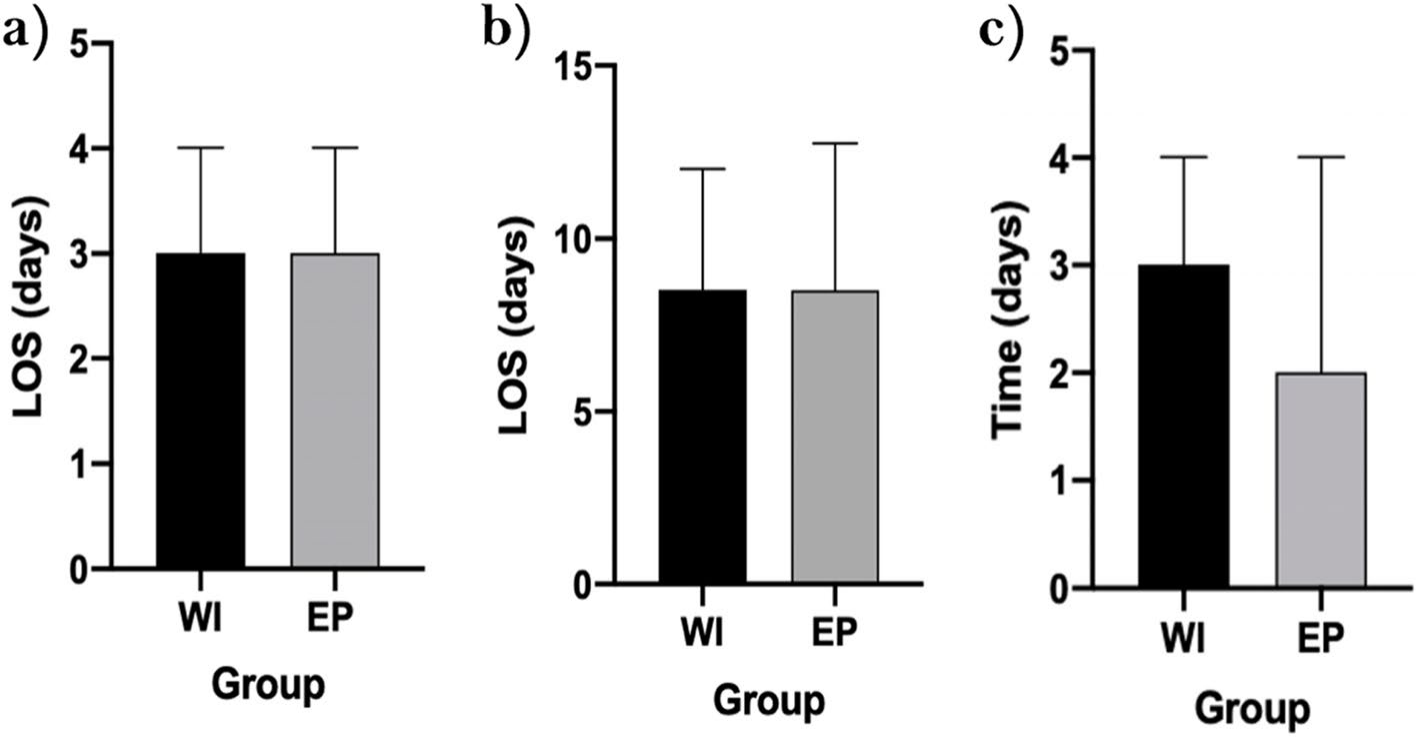
The median HDU and overall length of stay (LOS) (days), along with time (days) to eating and drinking **a**) LOS HDU, **b**) Overall LOS, **c**) Eating and drinking

The median length of stay (days) on HDU was 3 (IQR 2-4) for both the WI and EP groups (*p=0.354*). Furthermore, the overall length of stay (days) was 8 (IQR 7-12) for WI patients, compared with 8 (IQR 7-12.25) for EP patients (*p=0.846).* The time to eating and drinking (days post-op) was found to be 3 (IQR 2-4) in the WI arm and 2 (IQR 1-4) in the EP arm (*p=0.455*) (Fig 2). The complications noted for IIIb-IVb included two anastomotic leaks, one liver abscess, one bowel perforation, two intraabdominal collections and one case of sepsis. There was also no record of any specific complications associated directly with epidural or wound catheter patients recorded. Of the class I complications, neither arm had patients that received fluid boluses due to post-operative hypotension, nor had respiratory depression.

### Additional analgesia

**Fig. 3:**
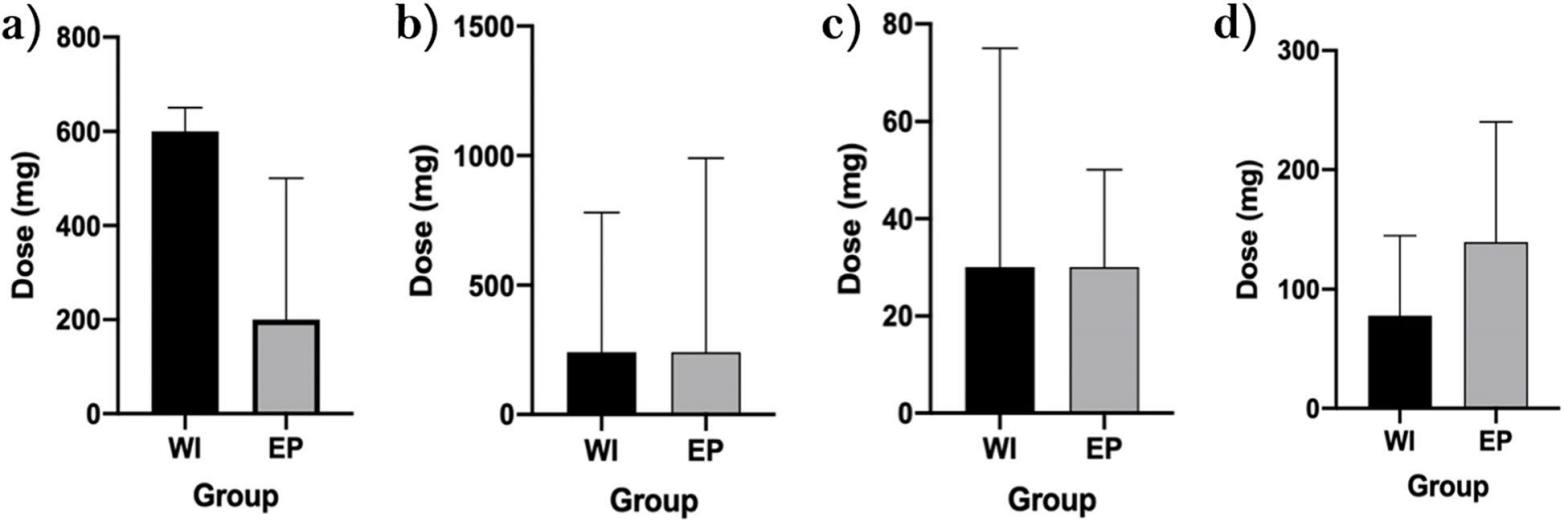
Median amount or oral and intravenous opioids taken post-operatively for both the WI and EP arm **a**) Tramadol, **b**) Codeine, **c**) Oromorph, **d**) IV PCA

With regards to oral opioids, patients in both the WI and EP group received either oromorph, codeine and/or tramadol on a relative needs-basis, during the post-operative period. The median amount of oromorph (mg) given was 30 (IQR 10-65) for WI patients and 30 (IQR 10-45) for EP patients (*p=0.829*). Similarly, the median amount of codeine (mg) given was 240 (IQR 52.5-540) within the WI group and 240 (IQR 12-960) within the EP group (*p=0.531*). Finally, the median amount of tramadol (mg) taken by patients was 600 (IQR 200-600) in the WI arm and 200 (IQR 100-500) in the EP arm (*p=0.073*) (Fig 3).

When analyzing the use of intravenous opioid analgesia in the post-operative period, it was found that 35/35 (100%) patients in the WI group received concurrent morphine sulphate (mg) through PCA. In the EP arm, 18/75 patients received concurrent morphine sulphate (mg) through PCA. The median amount of total morphine (mg) given through PCA was 80.5 (45-137.35) for WI patients and 139 (79.55-200.5) for EP patients (*p=0.137*) (Fig 3).

### Wound infusion +/- intrathecal analgesia

Within the WI arm, 24 patients received a pre-operative spinal injection (diamorphine, mcg) as well as post-operative PCA (WI-SP), with 11 patients receiving *only* post-operative PCA (WI-PCA). Operation duration (mins) was 210 in the WI-SP group and 196 in the WI-PCA group *(p=0.750)*

Intraoperative bleeding (ml) was 300 in the WI-SP group and 325 in the WI-PCA group (*p=0.975*). The amount of tramadol (mg), codeine (mg) and oromorph (mg) taken post-operatively was 200, 60 and 32.5 for the WI-SP group, respectively and 650, 420 and 60 for the WI-PCA group respectively (*p=0.298, p=0.890, p=0.776*). The amount of IV-PCA (morphine sulphate, mg) was 75 for the WI-SP arm and 101.5 for the WI-PCA group (*p=0.717*). The length of time (days) spent on HDU was 3 in the WI-SP group and 3 in the WI-PCA group (*p=0.973*), with overall length of stay (days) is 8 in both WI-SP and WI-PCA groups (*p=0.977*). Time to eating and drinking (days) was 3 in the WI-SP arm and 2 in the WI-PCA arm (*p=0.349*) (Table 3).

**Table 3:** Secondary intra-operative and post-operative comparison for wound infusion catheter patients with and without spinal analgesia pre-operatively

Patient data	WI -SP ***(n = 24)***	WI-PCA ***(n = 11)***	***P-value***
**Intraoperative data**			
Operation duration (mins)	210	196	*0.750*
Blood loss (ml)	300	325	*0.975*
Opioids given (mg)			
IV PCA	75	101.5	*0.717*
Tramadol	200	650	*0.298*
Codeine	60	420	*0.890*
Oromorph	32.5	60	*0.776*
Post-operative recovery (days)			
LOS HDU	3	3	*0.973*
LOS overall	8	8	*0.977*
Eating and drinking	3	2	*0.349*

## Discussion

This study aimed to assess the impact of specific analgesic modalities on overall patient recovery, assessing post-operative length of stay, commencement of oral diet, complications and amount of opioids given post-operatively, for patients who underwent open hepato-pancreato-biliary surgery.

Due to the common adverse effects of copious systemic opioids, such as respiratory depression, efforts have been made to develop peri-operative analgesia which minimizes patient exposure to intravenous opioid pain relief. The development of epidural analgesia has been shown to reduce these risks [12], but isn’t without its own drawbacks, such as; high failure rates (~30%), post-operative hypotension (associated with morbidity due to excessive intravenous fluids) [16], increased anaesthetic time [17] and coagulopathy-related complications in HPB surgery specifically [6, 7].

### Length of stay

A clear indicator of the rate of post-operative recovery was that of length of patient stay, both in HDU and overall. Our results highlight that there is no difference in the length of HDU stay between the WI-SP, WI-PCA or EP groups. This is in concordance with other studies in the literature, that show despite WI patients requiring less intensive monitoring than EP patients, HDU length of stay (days) was not significantly different (mean 1.3 vs 1.8, respectively) in patients undergoing liver resection [18].

Furthermore, our results show the overall length of stay did not differ between WI or EP arms; suggesting one modality does not confer an advantage with regards to overall post-operative recovery. This finding has been compounded by some studies found in the literature yet is contrasted to others. One RCT found there was no difference overall length of stay between WI or EP groups for patients undergoing liver resection [18], whereas another RCT found there was a reduced length of stay (days) in continuous wound infusion patients compared to those with epidural analgesia (4.5 and 6, respectively {*p= 0.044*}) [5].

### Commencing oral diet

Post-operative ileus is often an inevitable consequence of surgery and an undesirable effect that is further compounded by excessive use of intravenous opioids [19].

One particular study found that the use of thoracic epidural analgesia (TEA) accelerated GI motility post-operatively compared with IV PCA, with differences more pronounced on post-operative day (POD) 3 [19]. Our analysis of post-operative eating and drinking again found there to be no significant difference between the WI-SP, WI-PCA or EP groups with respect to time taken to begin oral diet. These data suggest no difference in rate or return to normal GI function (an important requirement for patient discharge) between aforementioned analgesic modalities.

### Additional opioids & pain management

Reducing post-operative pain is paramount in optimizing patient recovery and maximizing comfort during their hospital stay. Other studies in the literature have mainly used subjective pain scales to assess the efficacy of analgesic modalities [5, 18], but the retrospective nature of this study meant this information was unobtainable. Instead, we collected objective data based on amount of oral and intravenous opioids given to patients on a PRN basis, which found insignificant differences between the WI and EP groups in the amount of oral opioids taken post-operatively.

Although one particular systematic review of RCTs found wound catheters to provide improved analgesia [9], other literature looking at subjective pain scores have found epidural analgesia to be superior to continuous wound infusion on each day post-op [5, 18]. Nonetheless, even if this was the case with the patient data analyzed in this study, it did not translate into a significant oral opioid consumption in the WI group. Whilst we did not know the baseline analgesic medications taken by each patient prior to surgery, none were known to have chronic pain.

Further to oral opioids, the amount of IV morphine sulphate (PCA) administered between each group was compared. The use of IV PCA in patients with an epidural catheter has previously been defined as epidural failure [20]. From our data set, 24% (n=18) of EP patients received PCA, which is in concordance with previously documented EP failure rates of 20-30% [14, 16, 17]. Although insignificant, the median amount of IV morphine sulphate administered to the EP group was higher than in the WI group (WI-SP and WI-PCA).

### Overall complications

Using the Clavien-Dindo classification of post-operative complications, most patients in both the WI and EP groups had only class 1 complications, followed by class II. Of the class I complications, neither arm had patients that received fluid boluses due to post-operative hypotension, nor had respiratory depression. In contrast to this, other studies in the literature have found more complications in patients receiving epidural analgesia compared to wound-infusion groups, with one RCT showing a significant difference in vasopressor requirement on POD 0 (*p=0.001*) and 1 (*p=0.021*) in EP patients when compared with WI patients [14]. Whilst this study did not demonstrate any differences in hypotension-related treatment or complications between the two arms, it is a well-recognized sequela of epidural analgesia. Whilst the scope of this paper did not include direct comparison of peri-operative blood pressure and fluid balance, it is an important area that needs addressing and quantifying in future studies.

Similarly, a non-comparative study looked at the rate of hypofunction (inadequate pain relief) and hyper-function (hypotension or oliguria) of EP patients after pancreatectomies [20], finding hypofunction in 35% and Hyperfunction in 14% (combined complication rate of 49%) [20].

As 69% (n=24/35) of the WI patients analyzed received a pre-operative spinal injection, it is important to compare both the efficacy and complications of epidural and spinal analgesia. A retrospective study analyzing post-op complications after open HPB surgery showed that of 51 patients receiving epidural analgesia, 41% (n=21) experienced post-operative hypotension, compared 9% (n=7/79) of patients receiving intrathecal morphine [13]. Furthermore, the quality of intrathecal morphine (ITM) was found to be noninferior to EP, with reduced hospital stay and favourable cost [16]. It has also been found that the efficacy of analgesia produced via spinal injection is superior to IV PCA alone, while concurrently reducing IV morphine consumption [13]. In our data set, although not significant (*p=0.137*), the median IV morphine administered in the EP group was greater than that for the WI group overall, despite every patient within the WI arm receiving IV PCA. Whilst epidural failure did not translate to increase post-operative complication rate in this study, such a reportedly high failure rate highlights the need to explore alternative analgesic options.

## Conclusions

Although epidural analgesia is currently the gold standard for patients undergoing open abdominal surgery, there are rare incidences of life-threatening complications [1, 3] along with common complications such as PONV and hypotension [4]. This retrospective analysis found WI (+/- SP) to be non-inferior to EP with regards to length of stay, commencement of oral diet, overall post-operative complications and amount of oral and intravenous opioids given. For those patients in which EP is refused or contraindicated, WI-PCA (+/- SP) is a viable alternative.

### Limitations

This study has potential limitations which should be noted. Due to the retrospective nature of this study, the quality of pain relief could not be subjectively assessed using recognized pain scales, which would have been a more accurate representation of the analgesic efficacy of both modalities. The fact that the choice between the two analgesia methods was clinician dependent (as opposed to randomly assigned) is another potential confounding factor. Finally, although none of the patients were known to have any pre-existing chronic pain, we did not know the baseline pain medication requirements for patients pre-operatively.

## Data Availability

All data generated or analysed during this study are included in this published article [and its supplementary information files].

## Abbreviations

PCA: Patient controlled analgesia
WI: Wound infusion catheter
EP: Epidural
SP: Spinal
BMI: Body mass index
ASA: American Society of Anesthesiologists classification
HDU: High dependency unit
POD: Post-operative day
